# Introduction of multi-dose PCV 13 vaccine in Benin: from the decision to vaccinators experience

**DOI:** 10.1186/s12889-020-09326-9

**Published:** 2020-08-08

**Authors:** Daleb Abdoulaye Alfa, Roch A. Houngnihin, G. Patrick Ilboudo, Naomi Dick, Landry Kaucley, Téné-Alima Essoh

**Affiliations:** 1 Laboratoire d‘Anthropologie Médicale Appliquée (LAMA), University of Abomey-Calavi, Cotonou, Benin; 2 Maladies Infectieuses et Vecteurs - Ecologie, Génétique, Evolution et Contrôle (MIVEGEC), Institut de Recherche pour le Développement (IRD), Centre National de la Recherche Scientifique (CNRS), Montpellier, Cotonou, Benin; 3 Agence de Médecine Préventive (AMP) Ouagadougou, Ouagadougou, Burkina Faso; 4 Agence de Médecine Préventive Bureau Régional Afrique (AMP), Abidjan, Côte d’Ivoire; 5 Agence Nationale pour la Vaccination et les Soins de Santé Primaires du Benin, Cotonou, Benin

**Keywords:** Pneumococcal conjugate vaccine, PCV13, Decision-making, Multi-dose vials, Single-dose vials, Vaccinator experience, Benin

## Abstract

**Background:**

In 2011, Benin introduced the 13-valent pneumococcal conjugated vaccine (PCV13), in a single-dose vial, into its Expanded Programme for Immunisation (EPI) with support from Gavi. In April 2018, with the support of the Agence de Médecine Préventive Afrique (AMP) and other technical and financial partners, the single-dose vial was transitioned to a four-dose vial. Here we describe the decision-making process and the experience of the vaccinators during the change.

**Methods:**

We carried out semi-structured, individual interviews with 61 participants individuals involved in the EPI: 7 from central level, 5 from regional level, 7 from township level and 42 from district level. The interviews were recorded and transcribed, and the information categorised, using Nvivo software, and then analysed.

**Results:**

The Inter-agency Coordination Committee (ICC), the Benin National Advisory Committee for Vaccines and Vaccination, (BNACVV) and the World Health Organisation (WHO) (i.e., the traditional governance structures involved in vaccination decisions) were not involved in the decision to change to the four-dose vial for PCV13. The decision was taken by the EPI, supported by Gavi.

The vaccination errors observed in the first months following the change in presentation were due to the absence of guidelines for changes in vaccine presentation and the central-level actors’ perception that it was ‘*only a change in the vial*’, and therefore that the communication and training for a new vaccine were not required since the vaccine itself and its administration mode were unchanged.

**Conclusions:**

It is important that the other countries eligible for Gavi support that are about to change to the multi-dose vial PCV13 presentation learn from Benin’s experience. The main lessons learned are that changes in the presentation of an established vaccine should follow the same process as the introduction of a new vaccine, and that all stakeholders involved in vaccines and vaccination should participate in the decision-making process and implementation.

## Background

*Streptococcus pneumoniae* (pneumococcus) is the most common cause of severe pneumonia and pneumonia-related deaths worldwide. Pneumococcal bacteraemia can result in sepsis with death in up to 20% of patients, and the mortality rate for meningitis in developing countries is 50%, with the highest rates seen for young children [1]. World Health Organisation (WHO) estimated that 476,000 (333,000 to 529,000) of the estimated 8.8 million annual deaths worldwide in 2008 among children under 5 years of age were attributable to pneumococcal infections [2].

In many countries, the routine use of pneumococcal conjugate vaccines has led to a dramatic decline in severe pneumonia [3]. In some regions, pneumococcal bacteraemia caused by serotypes targeted by the pneumococcal conjugate vaccine (PCV) has virtually disappeared, even in age groups not directly targeted by the vaccination programmes. In 2011, with the support of Gavi, Benin introduced the single-dose vial (SDV) presentation of the 13-valent pneumococcal conjugate vaccine (PCV13 SDV, Pfizer, New York City, USA) into its Expanded Programme on Immunisation (EPI).

A multidose vial presentation (MDV) of PCV13 (PCV13 MDV, Pfizer, New York City, USA) that contains four doses has been prequalified by WHO and is eligible for Gavi support and therefore available at a reduced cost per dose [4]. Benin introduced this new presentation into its EPI in April 2018 with the financial support of Gavi and the strategic and technical support from WHO, UNICEF (United Nations International Children’s Emergency Fund) and the Agence de Médecine Préventive bureau Afrique (AMP). Through its local and regional representation AMP participates alongside WHO, UNICEF and Gavi and the country’s vaccination structures, in the planning, coordinating and implementation of vaccination strategies.

Within the framework of the introduction of the PCV13 MDV, AMP’s technical support resulted in the implementation of an innovative ‘training of trainers’ programme, with financial support from Pfizer and technical consultation from WHO, that aimed to empower healthcare workers in the change to the novel presentation for the pneumococcal vaccine by providing them with an overview of pneumococcal disease and a refresher on the proper use and storage of vaccines delivered in multi-dose vials. The training programme involved cascade training and was based on workshops, ‘war games’, and role play. Key documents were provided for use by healthcare workers involved in the vaccine delivery process. The programme started with the identification of ‘master trainers’ from the various countries that were going to implement the change to the novel presentation of the pneumococcal vaccine who were trained and then were tasked to provide training in the countries using a cascade approach, down to the healthcare personnel involved in delivering vaccination.

The decision to change the presentation of the PCV13 vaccine was based on logistic and economic considerations because end-to-end supply chain storage capacity is a problem for the majority of countries eligible for Gavi support, which limits the introduction of new vaccines and hinders equitable access to vaccination. On average, PCV13 SDV occupies nearly half the supply chain storage capacity of all EPI vaccines [5, 6]. One advantage of an MDV presentation is that it occupies about half the space occupied by the SDV presentation. In addition, the cost of filling and labelling the MDVs is lower than for the SDVs, which translates into a lower cost per dose [7, 8]. Some disadvantages of an MDV presentation have been reported; for example, vaccine dose wastage may be a problem [5, 9, 10], which could be avoided by the correct application of the WHO recommended MDV policy [11].

**Fig. 1:**
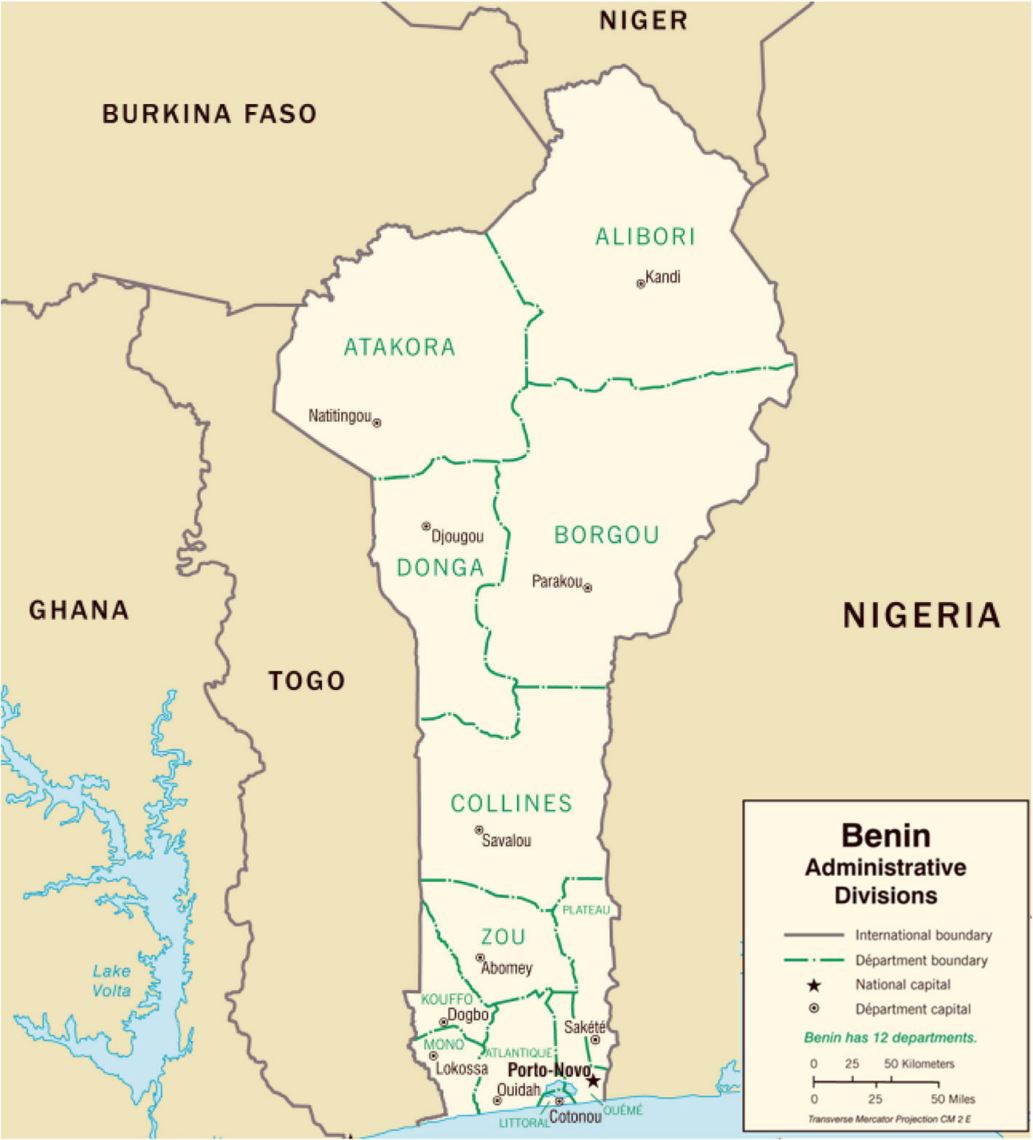
The Republic of Benin and its 12 administrative departments (adapted from Mapsland, https://www.mapsland.com/africa/benin/large-detailed-administrative-divisions-map-of-benin-2007)

The Republic of Benin is one of the countries that introduced the pneumococcal MDV into their EPI. It is located in West Africa and is bordered to the east by Togo, the west by Nigeria, the north by Burkina Faso and Niger, and in south by the Bight of Benin, in the Gulf of Guinea (Fig. 1). The country is divided into 12 administrative departments. French is the official language, but each ethnic group has its own language, which is also spoken. Benin has a national health care system that maintains hospitals in Cotonou, Porto-Novo, Parakou, Abomey, Ouidah, and Natitingou, in addition to medical dispensaries, maternity centres, and other small, specialized health care facilities in these and smaller towns. Financial aid from international organisations provides resources to compensate for a shortage of medical personnel and medications. The process for decision-making about the introduction of vaccines into the EPI in Benin is summarised in Additional File 1.

Here we describe and analyse the decision-making process and vaccinators’ experiences when a new presentation of an established vaccine was introduced into the EPI in Benin [4]. The lessons learnt from this experience could guide others considering changing the presentation of an existing vaccine.

## Methods

### Data collection methods

The methodology used was based on guidance developed by WHO for post-introduction assessments following the introduction of new vaccines [12]. In line with WHO’s guidance that recommends conducting the post-introduction assessment 6 to 12 months after introduction), the data collection took place 9 months after the multi-dose PCV13 vaccine had been introduced.

The qualitative evaluation was a component of the vaccine post-introduction assessment that explored the experiences with the change in vaccine presentation of those involved in the EPI. Data were collected via direct observations and semi-structured interviews [13]. Specific interview guides for central level and non-central level participants were developed for this study and were used for the semi-structured interviews to collect detailed information on the participants’ experiences [14]. The original guide was developed in French, but a translated English-language version is available in Additional File 2. The questions aimed to collect information about the experiences of various EPI actors with the change in vaccine presentation, the impact of the multi-dose PCV13 introduction on routine activities, the training received by health personnel for the multi-dose PCV13 administration, communication about the new vaccine introduction, as well as the expectations and recommendations of the various actors.

The study participants gave their consent for the interviews to be recorded. The interviews were conducted in French, when possible, but for participants who did not speak French, they were conducted in their local language and translated into French for analysis. The study protocol was approved by the Local Ethics Committee for Biomedical Research of the University Parakou in Benin.

### Recruitment of study participants

**Fig. 2:**
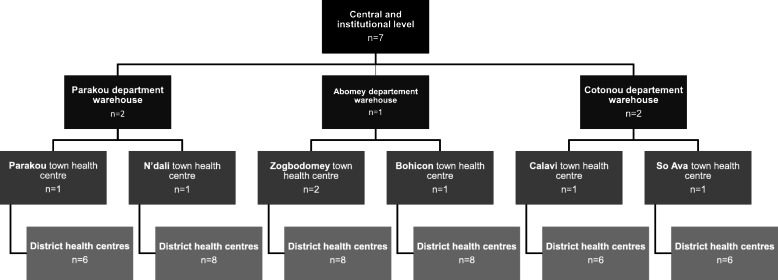
Summary of number of participants included at each level of the Benin health pyramid

Study participants were identified using a purposive sampling method to represent participants in the EPI who were at different levels of the Benin health pyramid. We included 61 key individuals at the central, regional, township and district levels, including representatives and partners of the WHO and UNICEF who provide technical support and advice for the EPI, the National Immunization Technical Advisory Group (NITAG) of Benin and the Inter-agency Coordination Committee (ICC) (Fig. 2). Healthcare professionals responsible for vaccination in each of the selected health centres were also interviewed.

### Choice of health zones and health centres

A retrospective survey using qualitative methods was conducted to investigate vaccinators’ experience relative to the change in PCV13 vaccine presentation. The health zones selected for data collection used either the PULL or PUSH strategy for vaccine distribution. In the PUSH approach, vaccines and injection supplies are distributed according to an allocation mode while in the PULL scenario they are distributed according to a requisition mode. The health zones selected were Abomey Calavi- Sô Ava (South, PUSH); Zogbodomey-Bohicon-ZaKpota (Centre, PULL); and Parakou-N’dali (North, PUSH). A total of 47 public health centres in 6 towns in these health zones were targeted (Table 1). The criteria for including these health centres were:

- accessibility of the health centre;
- the availability or not of a refrigerator in the health centre; and
- the involvement chief and assistant nurses in vaccination activities.

**Table 1:** Summary of the characteristics of Benin health centres and individuals included in the study

Department	Location / Health zone	Town	Health centre	Accessibility	Refrigerator availablein centre	Individual interviewed
Borgou	North-Benin/Parakou-N’dali	Parakou	Madina	Good	Yes	Assistant nurse
			Banikanni	Good	Yes	Assistant nurse
			Parakou	Good	Yes	Chief nurse
			Gannou	Good	Yes	Assistant nurse
			Zongo	Good	Yes	Assistant nurse
			Tourou	Good	Yes	Chief nurse
			Kpébié	Good	Yes	Assistant nurse
		N’Dali	Bori	Poor	Yes	Assistant nurse
			Sirarou	Good	Yes	Chief nurse
			N’Dali	Good	Yes	Chief nurse
			Témé	Poor	Yes	Assistant nurse
			DarNo	Good	No	Assistant nurse
			Gounin	Poor	No	Chief nurse
			Ouénou	Good	No	Assistant nurse
			Marégourou	Poor	No	Chief nurse
Zou	Centre-Benin / Zogbodomey-Bohicon-Za Kpota	Zogbodomey	Massi	Good	Yes	Chief nurse
			Zoukou	Good	Yes	Chief nurse
			Avlamé	Poor	Yes	Chief nurse
			Zogbodomey	Good	Yes	Chief nurse
			Canan	Poor	Yes	Assistant nurse
			Zoungbo-BogNo	Poor	Yes	Assistant nurse
			Dèmè	Poor	No	Chief nurse
			Koussoukpa	Poor	No	Chief nurse
		Bohicon	Bohicon I	Good	Yes	Chief nurse
			Bohicon II	Good	Yes	Chief nurse
			Sodohomey	Good	Yes	Chief nurse
			Saclo	Poor	Yes	Chief nurse
			Avogbannan	Good	Yes	Assistant nurse
			Gnindjazoun	Poor	Yes	Assistant nurse
			Passagon	Good	Yes	Chief nurse
			Ouassaho	Poor	No	Chief nurse
			Lissèzoun	Poor	Yes	Assistant nurse
Atlantique	South-Benin/Abomey/Calavi-So Ava	Abomey Calavi	Abomey-Calavi	Good	Yes	Chief nurse
			Akassato	Good	Yes	Chief nurse
			Glo	Good	Yes	Chief nurse
			Togba	Good	Yes	Assistant nurse
			Ouèdo	Good	Yes	Chief nurse
			Godomey	Good	Yes	Assistant nurse
		So Ava	So Ava	Poor	Yes	Chief nurse
			Véky	Poor	No	Chief nurse
			Ganvié	Poor	Yes	Chief nurse
			Gbéssou	Poor	Yes	Chief nurse
			Kinto	Poor	Yes	Chief nurse
			Houédo Aguékon	Poor	Yes	Chief nurse
			Ahomey Lokpo	Poor	Yes	Chief nurse

### Data collection

Four researchers conducted the interviews in the three selected health zones and at the central level from 6 to 28 February 2019. All four researchers collected data simultaneously in the Abomey-Calavi/Sô-ava health zone so that they could adjust the interview guides before going to the other two zones in pairs, following inductive qualitative methods [15, 16]. At the end of each day, the two pairs shared their data and potential new topics to be explored.

### Data analyses

After transcription and translation (when carried out in local languages) by experienced transcribers, the interviews were reviewed by the researcher who had conducted them before sharing them with the senior anthropologist. Based on the themes in the interview guides, the senior anthropologist carried out thematic sorting of all interviews using Nvivo software before conducting a content analysis. All information was analysed by category of meaning. In addition, the terms used by the interviewees to describe the problems studied and, additional themes identified by the researcher even if the interviewees did not take them into account were noted [17].

## Results

The results are organised in four sections. The first section describes the process and the role of various national and international actors in the introduction of the multi-dose PCV13 vaccine. The second section describes the preparatory activities preceding the introduction of the new vaccine presentation, including training and communication. The third section describes the real-world implementation of the vaccine, and the fourth describes the views of operational-level actors of the advantages and disadvantages of the switch from a single- to multi-dose PCV13 presentation.

### The decision-making process

#### A national decision advocated by external partners

The key stakeholders involved in decision-making for vaccine and immunization issues in Benin were interviewed to understand the process that led to the change from the PCV13 SDV to the PCV13 MDV presentation. Individuals from the National Agency for Immunization reported that the decision to change the presentation was proposed by UNICEF and Gavi and said they had accepted in the light of its potential advantages, particularly the impact on the cold chain storage capacity. In addition, they said they were told that Benin would be following the example of other countries in the region, such as Senegal. Others, from the National Agency for Immunization, explained that this was a deliberate choice in view of the country’s cold chain and storage capacity problems, particularly at the peripheral level. The choice was also seen as innovative, in anticipation of the introduction of new vaccines into the EPI.

#### Key actors not involved in decision-making process

We wanted to determine if the committees and structures from the technical and financial partners usually involved in immunization took part in the decision-making process. The process usually followed in Benin is described in Addition File 1. A respondent from the National Agency for Immunization said that a technical group, which is a subcommittee of the ICC, met to decide if the PCV13 presentation should be changed. The conclusions of this subcommittee were not recorded in the minutes, which prevented the decision-making process to continue until an ICC meeting was held. The respondent also mentioned a lack of leadership and ownership of decisions by national bodies, such as the ICC and NITAG, who only issue attestations about the country’s approval, preferring to follow Gavi. However, the respondent did not believe that this was an acceptable reason for not consulting the National Agency for Immunization about the change in presentation.

Another respondent from the National Agency for Immunisation said that the ICC was informed about the change in the PCV13 presentation, during a meeting when this was not on the agenda. The respondent said that they thought it was not necessary for the NITAG to be involved in the decision-making process for the presentation change, as it was not a new vaccine being introduced. These two committees were consulted in 2011 when the PCV13 SDV was first introduced in Benin; therefore, further consultation is optional, not mandatory. In addition, some actors at the central level said that consulting the NITAG is costly and time-consuming (6-months), and, therefore, not appropriate for urgent decisions.

Since UNICEF and Gavi had proposed to change the presentation, the sense of non-obligation to consult the NITAG and the ICC was reinforced. The change in the tetanus vaccine presentation happened under similar conditions, except that the ICC was consulted a posteriori. This respondent said:

“Ideally, we should have consulted the NITAG, but we were in a hurry because there was a deadline for all countries to change their presentation..." (National Agency for Immunisation Manager, Cotonou)

The contradictory versions about the involvement of the ICC and NITAG within the National Agency for Immunisation, the structure responsible for the coordination of vaccination activities, lead to us to interview some members of the ICC and NITAG directly. The members said that their committees had not been consulted and that they had become aware of the change through their involvement in National Agency for Immunisation. The participant from the NITAG said that, as of the date of the interview, none of the NITAG committee members had received official information about the change in PCV13 presentation. Although the participants from the NITAG and ICC said they were uncertain about the obligation of the National Agency for Immunisation to consult their committees, they were surprised that the committees that are responsible for vaccines and vaccination in Benin had not been officially informed about the change.

The WHO, another important stakeholder usually involved in the introduction of vaccines in the EPI, had also not been consulted. The WHO respondent said that they had been informed about the change by chance during a meeting that was not related to the PCV13 vaccine. Although they had been aware that a change was going to occur, they did not know the details about the date of the decision and its implementation. It was only when this present study was launched that the respondent was informed of the introduction of the vaccine.

"It's a management mode I don't understand, you can't manage like that! This is an important operation... Nothing can be done without involving the actors who are implicated in the intervention every day, particularly since there are not many actors..." (WHO respondent, Cotonou)

In addition, the relevant Ministry of Health departments, such as the National Directorate of Public Health or the Adverse Events Management Service, had not been informed or involved in the decision-making process that led to the change in presentation of the PCV13 vaccine.

### Preparation for PCV13 presentation change

#### Training cascade

Actors at central and departmental levels participated in a train-the-trainer session on multi-dose PCV13 administration, with the aim of cascading the training within their departments. The AMP were responsible for the train the trainer session, with financial aid via a non-restrictive educational grant from Pfizer, the vaccine manufacturer (Pfizer, New York City, USA). Logistics and vaccination department personnel at the central level were trained, as well as personnel from the Directors of Departmental Health and the Heads of the Departmental Public Health Services. The training course, which lasted two days and took place one month before the introduction of the PCV13 MDV, provided an update on WHO policy about the management of multidose vials, and details about pneumococcal diseases and vaccination.

The training cascade that should have followed should have involved the Heads of the Departmental Public Health Services running a 2-day training session for the Heads of the Departmental Division of Immunisation and Cost Recovery, who then organised a 2-day training session for the chief physicians and EPI managers in their health zone. The EPI managers should then have provided a one-day training session for all the health centre managers stationed in the area, who should have then trained the assistant nurses involved in vaccination activities in their respective health centres.

However, this was not possible due to lack of Gavi funding. A request for funding for activities related to the new PCV13 presentation should have been made at the central level by the National Agency for Immunisation, via the Gavi platform, but they had not been informed sufficiently early that they had to formally apply for this funding. Finally, a funding request was made one month before the arrival of the new presentation but at the time of the study interviews i.e. nine months after the introduction, the funding was still not available. The central-level respondents said that they felt that Gavi’s non-communication about the need to apply for funding suggested that it was not necessary to formally apply, but they agreed that the lateness of the grant application had contributed to the lack of funding for training.

However, the coordinating doctor in the Zogbodomey-Bohicon-Zakpota health zone took the initiative to request funding from UNICEF for the training cascade, without waiting for this to be organised centrally. Thus, all chief physicians and EPI managers in this health zone were trained for two days by the Heads of the Departmental Division of Immunisation and Cost Recovery, who had been trained by their Head of the Departmental Public Health Services. The EPI managers then provided one-day training for all the health centre managers stationed in the area, who then trained the assistant nurses involved in vaccination activities in their respective health centres. One of the trained EPI managers at the township level said that it would have been better if the introduction of the PCV13 MDV presentation had been a week after the training, while the information was still fresh, rather than the three to four weeks that actually occurred, since this would have perhaps avoided some of the reported errors (see below).

#### Information sources about the changed PCV13 presentation

At the central level, an administrative note announcing the change in presentation had been sent by the National Agency for Immunisation to all the Departmental Public Health Services, which had then been followed by a technical information sheet on the new presentation. The usual dissemination pathway whereby the Departmental Public Health Services cascade this type of information to the health zones, which in turn cascade the information to the health centres, did not happen in all departments, so that some, health zones did not receive the information.

The National Agency for Immunisation did not send the administrative note or technical information sheet to all of the central-level structures that are usually informed when a new vaccine is introduced. A respondent from the Vaccine Adverse Event Management Department of the Ministry of Health, told us that he had found out about the change during a meeting not dealing with vaccination. However, the respondent reported not being concerned since it was only a change in presentation, not the introduction of a new vaccine.

All the Heads of Departmental Public Health Services who attended the train-the-trainers session were expected to train their staff using a poster they received during training. The Heads of the Departmental Vaccination Divisions attended these staff training sessions, and, in addition, they received the training documents from the train-the-trainers session attended by the Heads of Departmental Public Health Services, to help them understand better. However, some Heads of Departmental Public Health Services suggested that it would have been better if the Heads of the Departmental Division of Vaccination and Cost Recovery had attended their train-the-trainers session, since they have a more direct contact with the town EPI managers than them.

The Heads of the Departmental Division of Vaccination and Cost Recovery have regular contacts with the central logistics department since they collect the vaccines for their departmental depots. Thus, they received information about the change of the PCV13 presentation informally from the logistics department, often before the Departmental Public Health Services had been informed.

The town EPI managers were informed about the change at different times by the heads of the vaccination division. Some were informed as soon as the heads of the vaccination division received the information because of their relationships. Others were informed during the training session given by the Heads of the Departmental Public Health Services to the staff, and some were only informed when the PCV13 MDV presentation was delivered.

During the interviews with town EPI managers and health zone logisticians, we were told that they had received information about an introduction of the PCV13 MDV presentation but not about the date. The arrival of the new presentation was confirmed to some only when the vaccine arrived at the warehouses. Some were informed, when they asked why they had received fewer boxes than usual, that the boxes contained vials with four doses of PCV13, not one. Some of the EPI managers expressed their surprise by saying:

"The new presentation was delivered without any prior information" (Town EPI Officer)

The local EPI managers and logisticians in turn informed the heads of the health centres, often during management committee meetings. Information also circulated on the WhatsApp forum in some districts. However, some heads reported that they had not received any information and only found out when they began vaccinating with PCV13 MDV. This was also reported by some assistant nurses, although others reported that they were informed by the town EPI managers when they went to collect the vaccine at the communal warehouse. In addition, some health centres use community contact points to collect vaccines, and some respondents reported that these contact points had received information about the change in presentation but had not passed it on.

### Implementation process for PCV13 multi-dose presentation

#### Presentation change: business as usual

The change in presentation of the PCV13 vaccine did not result in any specific activities for its implementation, other than the organisation of a train-the-trainers session. The stock sheets and vaccination activity sheets were not changed as they did not require any modification.

The management of the PCV13 SDV stocks at the central and peripheral levels was carried out normally until all were used. A few weeks of stock shortages were reported at central and departmental levels, without affecting the town and districts that still held their own stocks of PCV13 SDV.

The town EPI managers were not given any specific instructions for managing stocks of PCV13 SDV and MDV simultaneously. Most found themselves with the two presentations in their refrigerators and devised their own management approach. Some town EPI managers supplied PCV13 SDV to selected health centres and only supplied the new MDV presentation once the stock of SDV ran out. Some distributed single-dose vaccines at the town level until they ran out, while others distributed the new presentation to health centres without waiting for the stocks of single-dose vaccines to run out. These differences in distribution resulted in the PCV13 MDV presentation being introduced from April to June 2018, depending on the town.

No instructions were given for the follow-up of adverse events following immunisation (AEFI) related to the use of multi-dose PCV13, other than what is usually done. The respondents said that no AEFI had been reported yet but suggested that this may be due to the poor performance of the AEFI notification system.

Finally, a method for collecting information about any difficulties encountered by vaccinators at the central and intermediate levels had not been implemented. Difficulties in completing stock and inventory sheets encountered by the health centre staff were resolved by the EPI managers.

### Operational-level experience of the multi-dose PCV13 presentation

#### Direct experience of vaccinators

The two major issues raised by most caregivers were the lack of information and the lack of training about the PCV13 MDV presentation. One assistant nurse vaccinator said:

“It was after I had vaccinated five children that I noticed that there was some vaccine left in each of the five the vials, so I called the local EPI manager who said he had forgotten to inform me that there were 4 doses in each vial now*.*

Also many other vaccinators reported that they were only informed about the change when they received their vaccine supplies:

"...I received fewer boxes than usual. That's when I asked the Head of the Departmental Division of Immunisation and Cost Recovery who told me that it's now four doses in the vial" (local EPI manager)

The disparity in the level of information was amplified by the fact that the vaccinators said that the old SDVs and new MDVs were the same colour and size, with virtually no differences between them. Another problem mentioned by the vaccinators was the difficulty to complete stock management sheets. For example, the single-dose PCV13 vaccine was on the same stock management sheet as the pentavalent vaccine, which comes in 10-dose vials, so that, in the past, 10 vials of single-dose PCV13 vaccine were used for on vial of pentavalent vaccine, but 2.5 vials of the MDV presentation for PCV13 were used for each pentavalent vaccine vial, leading to confusion. Also, when both presentations were stored in the same refrigerators during the transition period, they found it difficult to complete the stock management sheets, since they recorded the number of vials used, not the number of doses administered.

Overall, the process of changing the PCV13 presentation generated stress among the vaccinators due to insufficient information, non-adapted management and non-adapted planning documents. Despite this, the participants interviewed recognised the significant advantages of the new presentation.

#### Increased risk of vaccine wastage

Some busy health centres have teams that go to villages and homes for vaccination sessions, and the returning teams often reintegrate the unused vials into the cold chain. Also, they often started more than one multi-dose vial of PCV13, and when they returned the vial to the refrigerator, they did not always write the opening date on the vial. The vaccinators said that it was not necessary to mark the date on open vials since they would be used within two days in busy health centres and one week in less busy health centres. However, the 4-dose vials of PCV13 MDV were found in the refrigerators of some health centres without the opening date marked, and so EPI managers would discard these vials during a control if there were any doubts about their opening date. This was reported at least 3 times in the first 2 months after the change in vaccine presentation in 15 health centers.

#### Logistical advantages

The main advantage of the multi-dose presentation reported by all the town health centres interviewed was that it takes up less space, which is important since some have storage problems. For example, one centre used two refrigerators for vaccines, one approved and the other not. But since the introduction of the multi-dose presentation, all vaccines can be stored in the approved refrigerator. In other town health centres, the reduced space needed made it possible to have a refrigerator exclusively for the town centres and another for the district health centres, making it easier to store vaccines in approved refrigerators. Furthermore, some towns the respondents said they only had to use one isothermal box instead of two to collect vaccines from the departmental warehouse because the PCV13 MDVs took up less room. In addition, the number of trips for the supply and distribution of supplies to health centres decreased, thus contributing to savings in time and fuel.

With the MDV presentation vaccinators reported that they only needed one isothermal vaccine box to transport the vaccine for advanced strategy vaccination sessions, which meant that the motorbike could take two people, instead of one. However, when a second isothermal vaccine box was needed, it was often not full, and therefore the vaccine vials could move, and sometimes they broke during transportation.

#### Convenient and timesaving

Most vaccinators said that not having to open a vial for each child made vaccination faster. In addition, the risk of tearing gloves when opening vials, which potentially exposes the vaccinator and children to risks, is reduced.

The vaccinators reported a significant reduction in the volume of waste with the MDV presentation. This is important since waste management in most health centres is not effective as either the incinerators are defective, or staff are not trained to operate the incinerators. As a result, incinerator use is generally limited and depends on the intervention of personnel from the health zone.

## Discussion

The decision to change to a PCV13 MDV presentation was taken by the vaccination and logistics departments of the National Agency for Immunization and Primary Health Care (ANV-SSP) in agreement with UNICEF and Gavi [18]. Although the actors usually involved in decisions about vaccines and vaccination, such as the ICC, the NITAG and WHO, were not consulted, they did not feel it was a problem, for this instance. Time and financial constraints supported this change, and this decision-making process can be understood in the context of absence of guidelines for changing vaccine presentations [19]. Sometimes the ANV-SSP has to omit steps when a decision must be taken quickly, for example when the tetanus vaccine presentation was changed, the ICC was consulted retrospectively [20]. In addition, the need for countries to respond to public health challenges by introducing new vaccines or changing their presentation based on the recommendation of their external partners could result in some actors in the vaccination system believing that the consultation of bodies such as the ICC and the NITAG is just a formality.

The preparation for the PCV13 SDV to MDV change involved the drafting of an administrative letter, the production of technical data sheets, the organisation of briefing sessions and the preparation of a train-the-trainers session. However, the information sheets did not always reach the vaccinators for whom they were designed. Briefing sessions were occasionally organized at the level of the departmental logistics centre rather than at the health facilities. In addition, some remote health centres do not have reliable access to Internet and, therefore, could not access information that circulated via WhatsApp, which has become the means of communication used in health zones. This situation led to disparity in the reception of information at the level of health centres.

The train-the-trainers sessions that were carried out by the AMP, and the non-receipt of the expected subsidy to support activities for the introduction of the new vaccine from GAVI probably also contributed to the laxity of those in charge of communicating to and training the EPI actors. In addition, the actors at central and departmental considered that since the PCV13 MDV was not a new vaccine, there would be no problems for the vaccinators in their daily work.

The absence of central guidelines or recommendations about how the PCV13 MDV presentation should be introduced resulted in each town EPI manager organising themselves as best they could. For example, the coordinating doctor in the Zogbodomey-Bohicon-Za Kpota health zone, who is a vaccinologist, took the initiative to obtain funding from partners without waiting for the cascade training to be organised centrally [21].

Our results showed that, regardless of the level of the actors (central or peripheral), the advantages of the multi-dose PCV13 presentation (i.e., space-saving in the cold chain, ease of vaccine transport and waste reductions) were unanimously accepted [5, 6]. In addition to these primary advantages, the vaccinators highlighted time-saving and safety as advantages for themselves and the children being vaccinated. However, many vaccinators were not informed about the new presentation, including the number of doses in the vial, when it was introduced and, as a result, many doses were discarded during the introduction period. A good communication strategy that targeted the vaccinators, even in the absence of training, could have avoided loss of dose and the frustration of vaccinators who did not feel considered.

Additionally, since PCV13 is administered at the same time as the pentavalent vaccine, which comes in a 10-dose presentation, it would be simpler and less stressful for vaccinators to have the PCV13 MDV in 5-dose or 10-dose presentations, to accompany the 10-dose vial of the pentavalent vaccine. With the 4-dose MDV, the vaccinators have to calculate how many PCV13 4-dose vials they need to match the 10-dose PENTA vials.

## Conclusion

The results from this qualitative study help to understand the decision-making process for the change from an SDV to an MDV presentation for PCV13, and the experiences of those involved in vaccination activities. The errors observed in the first months following the change from a single-dose to a multi-dose presentation of PCV13 were due to multiple reasons: first, the absence of guidelines for changes in the vaccine presentation; and second, the central-level actors’ perception that it was only a change in the vial and that the vaccine and its administration were unchanged. Taken together, this led to the belief that the change to MDV did not require the same of level of communication and training as a change to a new vaccine.

The main lessons were that changes in vaccine presentation should follow the same process as the introduction of a new vaccine, and all stakeholders involved in vaccines and vaccination should participate in the decision-making process and implementation. It is important that the other countries that change form a SDV to a MDV vaccine presentation, eligible or not for Gavi support, learn from Benin’s experience.

## Supplementary information

**Additional file 1.** Benin Health System and Process for New Vaccine Inclusion in the Expanded Programme for Immunisation. Description of the health system in Benin and the decision process for the introduction of vaccines in the Expanded Programme for Immunisation.

**Additional File 2.** Interview guides: English translation. English translation of the interview guides used for interviewing healthcare workers and central level actors

## Data Availability

The datasets used and/or analysed during the current study are available from the corresponding author on reasonable request.

## Abbreviations

AEFI: Adverse Events Following Immunisation
AMP: Agence de Médecine Préventive
ANV-SSP: National Agency for Immunization and Primary Health Care
BNACVV: Benin National Advisory Committee for Vaccines and Vaccination
EPI: Expanded Programme for Immunisation
ICC: Inter-agency Coordination Committee
MDV: Multi-Dose Vial
NITAG: National Immunisation Technical Advisory Group
PCV: Pneumococcal Conjugate Vaccine
SDV: Single-Dose Vial;
UNICEF: United Nations International Children’s Emergency Fund
WHO: World Health Organisation
