# Supplementary material for: Introduction of multi-dose PCV 13 vaccine in Benin: from the decision to vaccinators experience
Source: BMC Public Health. 2020 Aug 8;20:1216. doi: 10.1186/s12889-020-09326-9 (PMC7414647; doi:10.1186/s12889-020-09326-9)
Supplement: Supplementary file 1 — Additional file 1. Benin Health System and Process for New Vaccine Inclusion in the Expanded Programme for Immunisation. Description of the health system in Benin and the decision process for the introduction of vaccines in the Expanded Programme for Immunisation. [file 12889_2020_9326_MOESM1_ESM.pdf]

Benin Health System and Process for New Vaccine Inclusion in the Expanded Programme for Immunisation

Organisation of the Public Health in Benin

The public health system in Benin is a decentralized pyramidal structure with a central level consisting of the Cabinet of the Ministry of Public Health, within the Ministry of Health and the central and technical directorates, and an intermediate level with 12 departmental health directorates and their technical services. The peripheral level is composed of 34 health zones which bring together one or more administrative communes, the operational unit of the health system.

The Expanded Programme on Immunisation

The Expanded Programme on Immunisation (EPI), a World Health Organisation programme, was established in 1974 to develop and expand immunisation programmes throughout the world [1]. In 1977, the goal was set to make immunisation against diphtheria, pertussis, tetanus, poliomyelitis, measles and tuberculosis available to every child in the world by 1990. Currently the EPI also includes human papillomavirus (HPV), inactivated poliovirus (IPV), rotavirus, Japanese encephalitis, meningitis A, measles-rubella, cholera, yellow fever, and pneumococcal vaccines

In Benin, the EPI is implemented by the National Directorate of the Expanded Program on Immunization and Primary Health Care (NDEPI-PHC), the executing arm of State policy for immunization. It consists of four technical services: Immunization, Logistics, Primary Health Care and Cost Reimbursement. At the national level, the EPI is under the responsibility of the National Agency for Immunisation and Primary Health Care (NAI-PHC) that sits within the Minister of Health. Coordination is provided by a public health doctor, who is the National

Director for EPI and Primary Health Care. At the departmental level, the EPI coordination is provided by the Director of the Health Protection and Promotion Service, who is assisted by the Director of the Immunisation Division. At the health zone level, the EPI is coordinated by the Health Zone Committee (HZC) under the responsibility of the Health Zone Coordinating Doctor (HZCD). At the communal level, the EPI is coordinated by the Chief Commune Doctor seconded by an EPI Manager. At the local neighbourhood district level, the EPI activities are carried out under the guidance of the EPI Manager.

Process for Introduction of Vaccines in Benin

As Benin is eligible for Gavi funding, it can apply for support for its existing EPI and for the introduction and implementation of immunisation with new vaccines, including new vaccine presentations [2]. The Constitution of 11 December 1990 stipulates that 'the State shall protect the family and more particularly the mother and child'. Immunisation is free and compulsory for all children. The State does its utmost to mobilise sufficient resources to guarantee the procurement of vaccines and injection supplies. It relies on the expertise and facilities of its partners, in particular, UNICEF, which specialises in vaccine procurement.

The Inter-agency Coordinating Committee for EPI (ICC-EPI) provides the framework for partnership between the government of Benin and development cooperation agencies with a view to the programming, implementation, and follow-up of immunisation activities (Figure). The main ICC-EPI members are internal partners (government, NGOs, private sector and communities), and external partners consisting such as WHO, UNICEF, AMP, CDC and Gavi. The National Immunisation Technical Advisory Group, or NITAG, guide policy and programme decisions at country level. The NITAG plays a critical role in ensuring sustainable immunisation programmes by promoting evidence-based decisions and enable the country to take full ownership of their policies and immunisation programmes.

Figure: Outline of Structures Involved in Decisions and Implementation of Immunisation in Benin

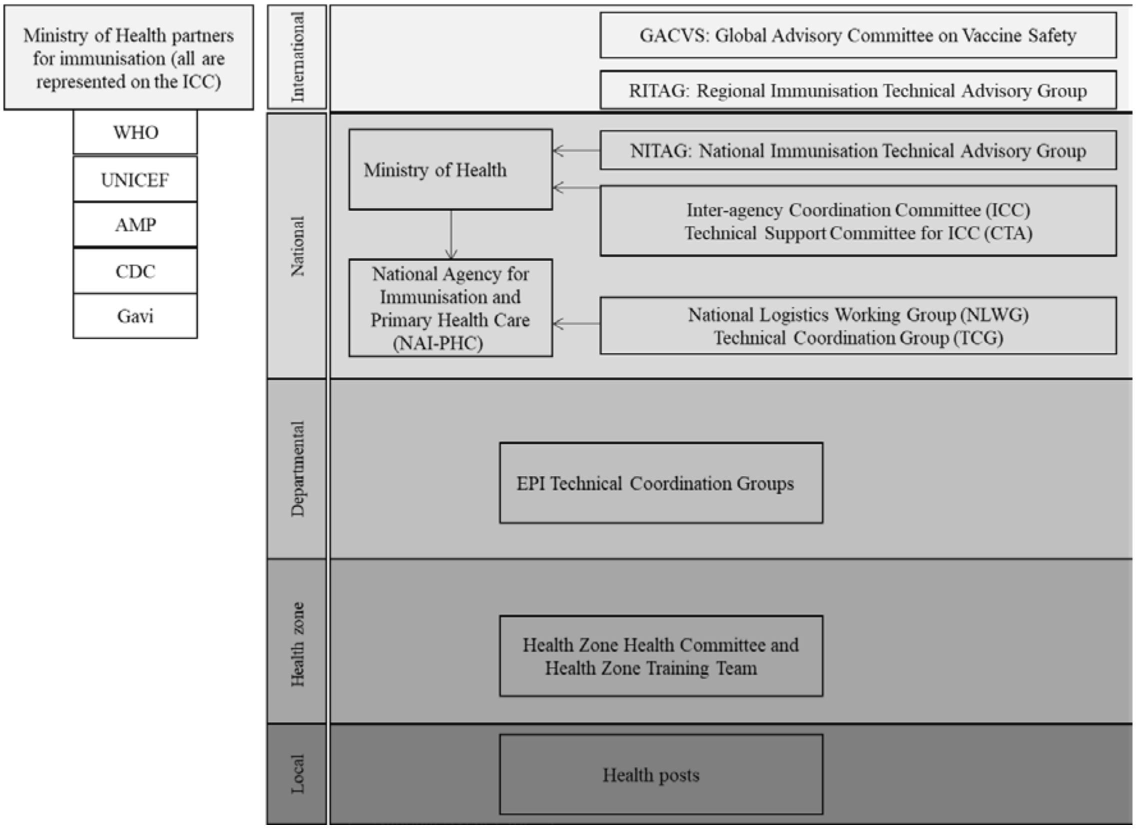

To initiate the process, the introduction of the new vaccine, needs to be planned in the State's Comprehensive Multi-Year Plan. The Health Ministry then officially consults the NITAG for their recommendations on the introduction of the vaccine. The NITAG takes into consideration the epidemiology of the vaccine-preventable disease and its economical impact and the efficacy and safety of the proposed vaccine to make its recommendations. The introduction of the vaccine is then presented to the ICC for their approval. If the vaccine is eligible, Gavi funding is requested, which takes about 18 months to obtain.

The NAI-PHC which sits in the Ministry of health is assisted by the National Logistics Working Group (NLWG) and the Technical Coordination Group (TCG) to implement the immunisation decisions taken by the Ministry of Health after consultation with the NITAG and the ICC and its

Technical Support Committee (ICC-TSC). The NAI-PHC provides them with the information and training necessary for the implementation of the new or modified vaccine. The Departmental EPI Technical Coordination Groups then liaise with the Health Zone Health Committees and Training Teams. These latter are then responsible for ensuring that the staff in the health posts are informed and trained.

References

1. WHO: The expanded programme on immunization [https://www.who.int/immunization/programmes_systems/supply_chain/benefits_of_immunization/en/] (2013). Accessed 29 July 2020.
2. Gavi: Comprehensive multi-year plans/Financial sustainability plans [https://www.gavi.org/country-documents/benin] (2020). Accessed 29 July 2020.
