# Supplementary material for: Introduction of multi-dose PCV 13 vaccine in Benin: from the decision to vaccinators experience
Source: BMC Public Health. 2020 Aug 8;20:1216. doi: 10.1186/s12889-020-09326-9 (PMC7414647; doi:10.1186/s12889-020-09326-9)
Supplement: Supplementary file 2 — Additional File 2. Interview guides: English translation. English translation of the interview guides used for interviewing healthcare workers and central level actors [file 12889_2020_9326_MOESM2_ESM.pdf]

Interview guides: English translation

IMPACT-SWITCH-PCV13-BEN project

Interview guide for healthcare workers

Impact of PCV13 MDV

How does the new PCV13 MDV presentation compared with the old one, regarding:

a. Organization of vaccination? (distribution of tasks, use of community relays, number of vaccination sessions, etc.?)

b. Vaccine preparation and anxiety for healthcare workers?

c. Time required for the preparation and administration of the vaccine?

d. Storage after opening the vial?

e. The rate of vaccine dose loss

f. Reporting (filling in vaccination records, forms and registers)

g. Safety i.e., adverse events following immunisation (AEFI)?

h. Missed opportunities for vaccination?

i. Logistics (storage and disposal in the refrigerator, transport for advanced strategy and waste treatment?

In your opinion, what are the other advantages and disadvantages of the new PCV13 presentation?

How does the new presentation compared with the old one regarding:

a. Routine vaccinations

b. Advanced remote fixed centre strategies

c. Advanced door-to-door strategies

What are your criteria for the efficacy of a vaccine? What do you think of the efficacy of the new presentation?

What do you think about the shelf life of the new vaccine? What do you think of the preservative? Do you think it has an impact on the vaccine's safety and efficacy?

Do you think this is just a change in vaccine presentation or a new vaccine? Why?

Training of healthcare workers

How were you informed about the introduction of the new presentation? Do you think the introduction went smoothly or was it problematic? Why?

Did you attend any training related to the introduction of the new presentation? What do you think about the training was organised? Did you attend the training before the introduction of the new presentation in the EPI?

What specific aspects were covered in the training? (injection safety, vaccine management, waste management, etc.)?

What do you think of the profile of the trainers? Do you think the trainer was competent? Why? Was it a cascade training? What do you think of the trainers and the training strategy?

Did you have practical sessions during the training? How long did the training last? Do you think you need additional training on a specific aspect? Why?

Did the training cover any other topics related to immunisation apart from the introduction of the new presentation?

Have all the centre agents involved in vaccination activities participated in this training? Why?

Do you have training materials for yourself or are they available in your centre? Why?

Were you monitored for the new presentation by someone in the health zone or at a central level ?

Expectations and Recommendations

Have you ever been involved in the introduction of new vaccines or change in vaccine presentation in the EPI? If yes, can you tell us more about this?

What would have been helpful for the introduction of this new presentation?

What do you think would have been the most efficacious strategies for introducing the new presentation?

Which vaccine presentation would you have liked to have to facilitate your activities? Would you have liked to have both presentations, i.e. single dose and multidose? Why?

Which vaccines do you think are the most appropriate for this form of presentation? Why?

Many more countries will be introducing this new vaccine presentation soon, as well as other vaccines. What did you learn from your experience and what advice would you give to other countries to enable a smooth introduction?

IMPACT-SWITCH PCV13-BEN project

Interview guide for central level actors

Process the change in PCV13 MDV presentation

1. What are the reasons behind the decision to change the presentation of PCV13? Which actor (s) brought about this change and what were their interests? Who are the actors who were involved in this process? And what role did each of them have to play?

2. Do you think that all the actors involved were in favour of this change? If not, who was reluctant and why?

3. How much time was there between the first proposal to change the presentation and its implementation in April? Why?

4. How was the PCV 13 presentation change process conducted? Describe the main steps:

- Training of healthcare workers: profiles and numbers of people trained, duration of training and its timing in relation to the introduction, profile of the trainer(s) and topics covered, theoretical or practical training, training materials consulted, if they existed)

- Communication and awareness of parents: existence of a communication plan and level of implementation, communication channels used, and key messages conveyed

- Monitoring: frequency and profile of monitors, timing of monitoring in relation to the introduction, lessons learned from monitoring.

Is there a planning document that guided the introduction of PCV13 MDV? If so, can we have a copy?

5. Do you think that this planning document was respected in the implementation? Why?

6. In your opinion, what are the requirements for the introduction of a new vaccine in the EPI in Benin? Do you think that these requirements were met in the case of PCV13 MDV? Why?

Impact of PCV13 MDV

1. What problems do you think the PCV13 MDV solves (organisation of vaccination, preparation for vaccination and anxiety for the nursing staff, time for preparation and administration of the vaccine, storage after opening, rate of vaccine dose loss, reporting issues, safety issues such as AEFI, missed immunisation opportunities, waste management, logistics issues? Can you provide details?

2. What new problems do you think the PCV13 MDV creates in the EPI (organisation of vaccination, preparation for vaccination and anxiety for healthcare staff, time for preparation and administration of the vaccine, storage after opening, rate of vaccine dose loss, reporting issues, safety issues such as AEFI, missed immunisation opportunities, waste management, logistics issues? Can you provide details?

3. What do you think are the criteria for a good vaccine? What do you think of the presentation of a vaccine? Is it an important element or just a detail ? Why?

4. How does the new vaccine compare with the old one regarding:

- Routine vaccinations
- Advanced remote fixed centre strategies
- Advanced door-to-door strategies

5. What do you think about the shelf life of the new vaccine? What do you think of the preservative? Do you think it has an impact on the vaccine’s safety and efficacy?

Expectations and Recommendations

Have you ever been involved in the introduction of new vaccines or change in vaccine presentation in the EPI? If yes, can you tell us more about this?

What would have been helpful for the introduction of this new presentation?

What do you think would have been the most efficacious strategies for introducing the new presentation?

Which vaccine presentation would you have liked to have to facilitate your activities? Would you have liked to have both presentations, i.e. single dose and multidose? Why?

Which vaccines do you think are the most appropriate for this form of presentation? Why?

Many more countries will be introducing this new vaccine presentation soon, as well as other vaccines. What did you learn from your experience and what advice would you give to other countries to enable a smooth introduction?
